# Exercise Training during Normobaric Hypoxic Confinement Does Not Alter Hormonal Appetite Regulation

**DOI:** 10.1371/journal.pone.0098874

**Published:** 2014-06-02

**Authors:** Tadej Debevec, Elizabeth J. Simpson, Ian A. Macdonald, Ola Eiken, Igor B. Mekjavic

**Affiliations:** 1 Department of Automation, Biocybernetics and Robotics, Jozef Stefan Institute, Ljubljana, Slovenia; 2 MRC/Arthritis Research UK Centre for Musculoskeletal Ageing Research, University of Nottingham Medical School, School of Life Sciences, Queen's Medical Centre, Nottingham, United Kingdom; 3 Department of Environmental Physiology, Swedish Aerospace Physiology Centre, Royal Institute of Technology, Stockholm, Sweden; University College London, United Kingdom

## Abstract

**Background:**

Both exposure to hypoxia and exercise training have the potential to modulate appetite and induce beneficial metabolic adaptations. The purpose of this study was to determine whether daily moderate exercise training performed during a 10-day exposure to normobaric hypoxia alters hormonal appetite regulation and augments metabolic health.

**Methods:**

Fourteen healthy, male participants underwent a 10-day hypoxic confinement at ∼4000 m simulated altitude (F_I_O_2_ = 0.139±0.003%) either combined with daily moderate intensity exercise (Exercise group; *N* = 8, Age = 25.8±2.4 yrs, BMI = 22.9±1.2 kg·m^−2^) or without any exercise (Sedentary group; *N* = 6 Age = 24.8±3.1 yrs, BMI = 22.3±2.5 kg·m^−2^). A meal tolerance test was performed before (Pre) and after the confinement (Post) to quantify fasting and postprandial concentrations of selected appetite-related hormones and metabolic risk markers. ^13^C-Glucose was dissolved in the test meal and ^13^CO_2_ determined in breath samples. Perceived appetite ratings were obtained throughout the meal tolerance tests.

**Results:**

While body mass decreased in both groups (−1.4 kg; p = 0.01) following the confinement, whole body fat mass was only reduced in the Exercise group (−1.5 kg; p = 0.01). At Post, postprandial serum insulin was reduced in the Sedentary group (−49%; p = 0.01) and postprandial plasma glucose in the Exercise group (−19%; p = 0.03). Fasting serum total cholesterol levels were reduced (−12%; p = 0.01) at Post in the Exercise group only, secondary to low-density lipoprotein cholesterol reduction (−16%; p = 0.01). No differences between groups or testing periods were noted in fasting and/or postprandial concentrations of total ghrelin, peptide YY, and glucagon-like peptide-1, leptin, adiponectin, expired ^13^CO_2_ as well as perceived appetite ratings (p>0.05).

**Conclusion:**

These findings suggest that performing daily moderate intensity exercise training during continuous hypoxic exposure does not alter hormonal appetite regulation but can improve the lipid profile in healthy young males.

## Introduction

High altitude sojourns are often associated with significant weight loss [1]. While several factors appear to be involved in this phenomenon, growing evidence suggest that appetite reduction plays an important role [2]. Indeed, decreased appetite and energy intake, collectively termed “altitude anorexia” is frequently reported during high altitude exposures and can induce negative energy balance, especially when coupled with increased levels of physical activity [3], [4].

Altitude-related appetite modulation is not entirely understood. Initially, the observed appetite reduction was only linked to acute mountain sickness [5]. However, the reported reductions in appetite, as well as energy intake, after altitude sickness symptoms subside imply other underlying mechanisms [1]. Among these, hormonal regulation might potentially play a role since several appetite-related gut hormones, and adipokines are affected by acute and/or prolonged hypoxic exposure [2], [6].

Acute hypoxia has been shown to reduce circulating levels of the appetite-stimulating hormone, ghrelin, both in the fasted [7] and fed state [6], [8], although the fasting ghrelin concentration reportedly returns to baseline levels after 7 and 49 days of continuous exposure [7], [9]. On the other hand, hypoxia has been shown to increase serum levels of the satiety-signaling hormone leptin, both acutely [7], [10], [11] and after 7 days at altitude [7]. Reports in the literature present contradictory findings with regard to both ghrelin and leptin responses to hypoxia [9], [12], presumably attributable to different experimental paradigms (hypobaric vs. normobaric hypoxia, rest vs. exercise and different hypoxic doses). Another potential explanation for the discrepancies between the outcomes is the fact that some studies measured total ghrelin [6], [7] while others measured the acylated ghrelin [8] known to be essential for ghrelin appetite-stimulating effects [13]. To date, the contribution of other potential anorexigenic hormonal modulators that can be altered by hypoxic exposures such as peptide YY (PYY) [8] and glucagon-like peptide-1 (GLP-1) [10], has received less attention. Hence, their role and contribution to altitude-related appetite reduction is currently unclear.

Similarly to hypoxia, acute or chronic exercise significantly modulates hormonal appetite regulation [14]. Acute exercise transiently suppresses appetite, while chronic exercise training typically results in an augmented appetite leading to a restoration of the exercise-induced energy imbalance [15]. Although the exact mechanisms of these exercise-induced acute appetite alterations are unclear, reductions in ghrelin and increases in PYY and GLP-1 have been proposed as the key underlying factors [15], [16]. Long-term studies have also indicated that chronic exercise training can improve appetite control system sensitivity [17] and augment fasting and postprandial satiety sensations [18]. While combining hypoxia and exercise is mostly used by athletes aiming to improve performance [19], it is gaining popularity as a novel modality for treatment of obesity with related comorbidities [2], [20], [21]. It has been suggested that hypoxia and exercise synergistically enhance metabolic health, since both individually up-regulate glucose, glycolysis and satiety related genes through hypoxia-inducible factor-1 stabilization [22]–[24]. Training under hypoxic condition has already been shown capable of improving metabolic status and glycemic control in healthy individuals [25], [26] and clinical populations [27]. However, the potential additive effects of exercise during prolonged hypoxic exposure on hormonal appetite regulation and metabolic risk factors have, to our knowledge, not yet been investigated. Only one study to date investigated the effects of rest and exercise in acute (7-hrs) hypoxia on specific appetite regulating markers, and noted that both hypoxia and exercise reduce hunger and ghrelin concentrations [8]. Given the potential application of hypoxia and exercise for improving metabolic health and fighting the escalating obesity pandemic, their combined effects on appetite regulation should be further elucidated. Moreover, the potential contribution of physical exertion to hormonal appetite modulations observed during prolonged high altitude sojourns is currently unresolved.

Accordingly, this study sought to determine the acute and prolonged effects of continuous normobaric hypoxic exposure and exercise training on hormonal appetite regulation and select metabolic risk factors. The participants underwent 10 days of normobaric hypoxic confinement, either with daily moderate-intensity exercise training, or without any exercise. It was hypothesized that the addition of exercise training to continuous hypoxia would result in further reduction of appetite, through a synergistic effect of hypoxia and exercise. We additionally hypothesized that performing exercise training during continuous hypoxia would further augment select indices of metabolic health.

## Materials and Methods

### Ethics statement

The present study was approved by the National Committee for Medical Ethics at the Ministry of Health of the Republic of Slovenia and conformed to the guidelines of the Declaration of Helsinki. The participants were informed in detail about the experimental procedures as well as the potential risks involved, and signed a written informed consent prior to the start of the study.

### Participants

Potential participants were invited to take part in this study through local advertisement. Exclusion criteria included chronic diseases, smoking, hematological, pulmonary and kidney disorders, use of any hormonal, metabolic or orexigenic/anorexigenic medications as well as any dietary manipulations within the last year. Applicants who had recently (≤1 month) been exposed to normobaric or hypobaric hypoxia (above 2000 m) were also ineligible to participate. Following initial laboratory screening and medical examination, 16 healthy males, all recreationally active low altitude residents (<500 m), were selected and equally assigned, in a randomized manner, to either the Exercise or the Sedentary group. As a result of medical issues during the confinement period (appendicitis, adaptation problems), two participants from the Sedentary group had to be withdrawn from the study. Consequently, eight participants in the Exercise (age  = 25.8±2.4 yrs, height  = 179.1±3.1 cm; BMI  = 22.9±1.2 kg·m^−2^, % body fat  = 22.8±6.2, peak O_2_ uptake in normoxia (VO_2peak_)  = 42.6±6.1 mL·kg^−1^·min^−1^) and six participants in the Sedentary group (age  = 24.8±3.1 yrs, height  = 177.7±3.5 cm; BMI  = 22.3±2.5 kg·m^−2^, % body fat  = 21.9±4.7, VO_2peak_  = 42.2±5.0 mL·kg^−1^·min^−1^) finished the study and only their data is reported in this paper.

### Experimental design

**Figure 1:**
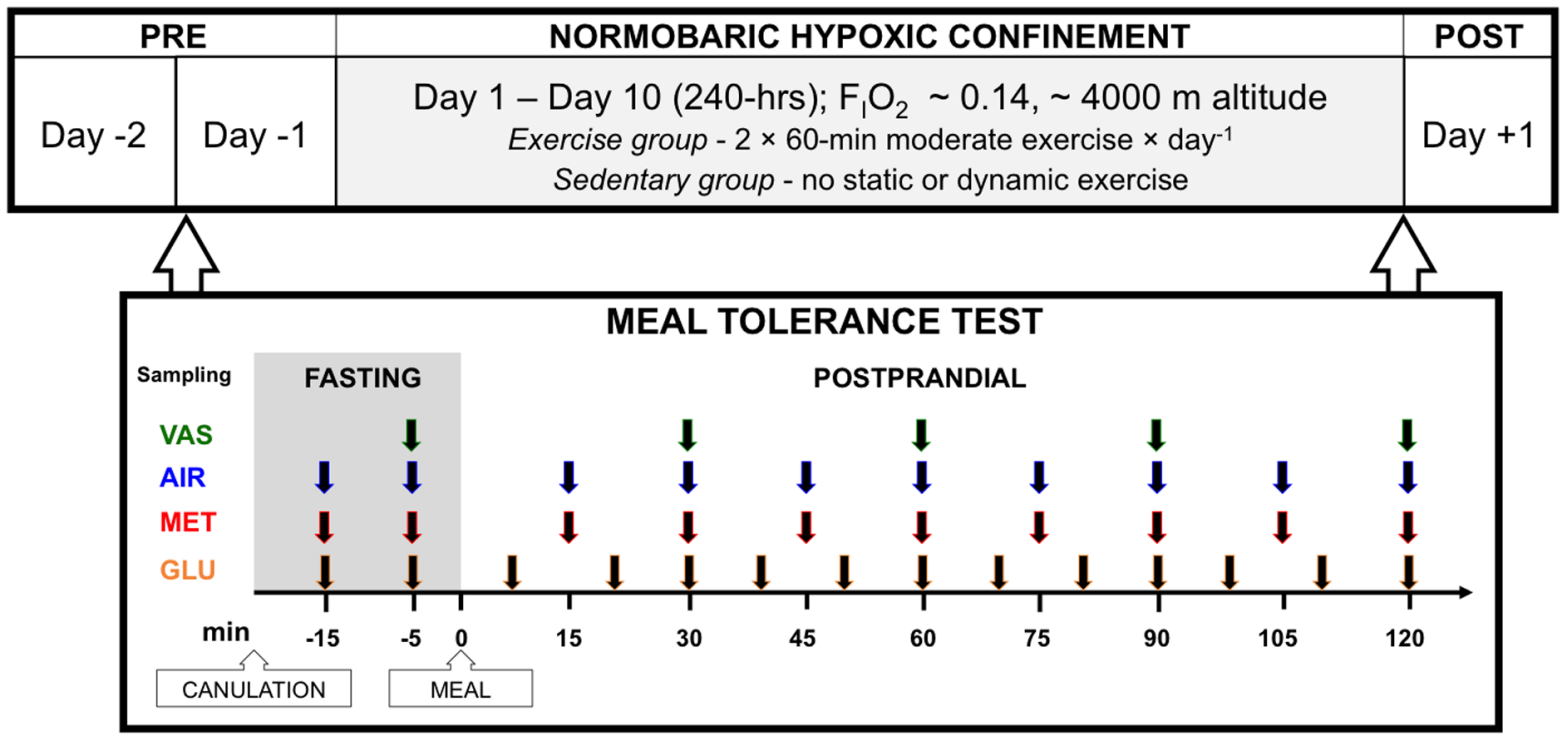
Study protocol and meal tolerance test outline. The meal tolerance test (MTT) was performed in the morning, one day before (Day -1) and just prior to exiting the 10-day confinement (morning of Day +1). F_I_O_2_, Fraction of inspired oxygen; VAS, Visual analogue scale for perceived appetite rating; AIR, Exhaled air sampling for determination of ^13^C isotope; MET, Blood sampling for determination of metabolic markers: total ghrelin, peptide YY, Glucagon-like peptide-1 and insulin at all samplings and leptin, adiponectin, free fatty acids, triacylglycerols, cholesterol, and catecholamines during fasting samplings only; GLU, Blood sampling for plasma glucose concentration determination.

This randomized, prospective trial was performed in the normobaric hypoxic facility of the Olympic Sports Center Planica in Rateče, Slovenia, situated 900 m above sea level. The study protocol, outlined in Figure 1, consisted of an initial testing period (Pre), a 10-day normobaric hypoxic confinement, combined either with daily moderate-intensity exercise training (Exercise group), or without any training (Sedentary group), and of an after testing period (Post). The participants were requested to arrive at the facility three days prior to the start of the protocol in order to familiarize them to the environment and to standardize their diet. They were accommodated in the hypoxic facility for the whole duration of the study protocol. A meal tolerance test (MTT) was performed at Pre and Post to determine the participants fasting metabolic status and postprandial metabolic responses. During the Pre testing period, the participants also performed graded exercise tests both under normoxic and hypoxic conditions to determine their VO_2peak_ and enable appropriate training intensity calculations.

### Experimental procedures

#### Normobaric hypoxic confinement

The participants were confined for 10 consecutive days to the hypoxic facility comprising ten double bedrooms, living area, dining area and connecting hallway. The confinement began on the morning of Day 1 and finished on the morning of Day +1 (Post), following the MTT, such that the cumulative individual hypoxic exposure time was exactly 240 hours. The normobaric hypoxic gas was generated and maintained using a Vacuum Pressure Swing Adsorption system (VPSA, b-Cat, Tiel, The Netherlands), that continuously delivered the oxygen depleted air into the confinement area to simulate an altitude of ∼4000 m (Fraction of inspired O_2_ (F_I_O_2_)  = 0.139±0.003; Partial pressure of inspired O_2_ (P_I_O_2_)  = 88.2±0.6 mmHg). Air samples were automatically drawn from each bedroom at 15-min intervals and analyzed by the VPSA system for O_2_ and CO_2_ fraction. The accumulated carbon dioxide level within the confinement area did not exceed 0.5% at any time. The environmental conditions on the whole floor were stable throughout the protocol (temperature  = 23.1±1.0°C; relative humidity  = 56±8% and ambient pressure  = 682±4 mmHg). The participants wore portable, ambient O_2_ analyzers with audible alarm (Rae PGM-1100, California, U.S.) at all times. Systolic and diastolic blood pressure (BP), heart rate (HR) and capillary oxyhaemoglobin saturation (SpO_2_) were measured each morning using an automated blood pressure cuff (Withings BP monitor; Withings, Les Moulineaux, France) and a finger oximetry device (3100 WristOx, Nonin Medicals, Minnesota, U.S.), respectively. Additionally, acute mountain sickness (AMS) symptoms and severity (Lake Louise score (LLS; 0–15)) were quantified daily using the self-assessment portion of the Lake Louise mountain sickness questionnaire [28]. AMS was defined as presence of headache and concomitant LLS of 3 or more.

#### Diet and fluid intake

The diet was individually adjusted, with the same macronutrient composition (expressed as percentage macronutrient contribution to total energy intake) used for all participants. The basal energy requirement of each participant was calculated using the Mifflin equation [29]. To account for the different energy expenditure requirements of the interventions, the values were subsequently multiplied by a physical activity level factor of 1.8 and 1.4 for the Exercise and Sedentary group, respectively. The targeted daily energy intakes were 3166±219 kcal for the Exercise and 2380±80 kcal for the Sedentary group. The menus were prepared using the web-based application Open Platform for Clinical Nutrition (OPKP (www.opkp.si), Jozef Stefan Institute, Ljubljana, Slovenia). The OPKP enables individual planning and monitoring of dietary intakes based on food tables. Five meals (breakfast, morning snack, lunch, afternoon snack and dinner) were served per day, with portion size adjusted according to the OPKP calculations to achieve the required energy intake for each individual. All meals were prepared in the facility kitchen by the staff of the Olympic Sport Centre. To provide each individual with the exact targeted intakes all food items were weighed on a precision (±0.1 g) scale (TPT 6C, Libela ELSI, Celje, Slovenia) prior to each meal. In case of any leftovers, the unconsumed food items were re-weighed and the outcome deducted from the initial weight to provide the actual food intake. The actual intakes were then used in final dietary analysis performed individually using the OPKP system (Table 1). The participants were encouraged to consume the meals provided in their entirety. They were allowed to drink water and unsweetened tea *ad libitum*, and were advised to drink at least 2 liters of fluid per day [30].

**Table 1:** Average daily nutritional intakes in the Exercise and Sedentary group during the 10-Day normobaric hypoxic confinement.

	Exercise group	Sedentary group
Energy intake (kcal)	2601±383 ##	2213±192 **
Carbohydrate (g)	344±106	286±60 *
Protein (g)	113±30	94±19 *
Fat (g)	84±23	76±20
Carbohydrate (% daily intake)	63±19	62±13
Protein (% daily intake)	21±5	22±4
Fat (% daily intake)	16±4	16±4
H_2_O (mL)	4872±1465	3283±1342 **
Fe (mg)	15.4±2.4	13.6±1.2 *
Na (mg)	4069±481	3428±477 *
Ca (mg)	1098±145	896±67 **

Values are means ± SD. * indicates significant differences compared to the Exercise group (* p<0.05, ** p<0.01); # indicates significant differences compared to pre-calculated intake (## p<0.01); H_2_O, Water; Fe, Iron; Na, Sodium; Ca, Calcium.

#### Baseline exercise testing

Details of the exercise testing and training protocol are presented elsewhere [31]. Briefly, all participants performed an incremental test to task failure on an electronically braked cycle-ergometer (Ergo Bike Premium, Daum electronics, Fürth, Germany) prior to the start of the confinement, to assess their baseline aerobic capacity. After an initial 2-min warm up at 60 W, the workload was increased by 25 W·min^−1^ until task failure (cycling cadence below 60 rpm). A calibrated metabolic cart (Quark CPET, Cosmed, Rome, Italy) was used for breath-by-breath gas exchange measurement. The average O_2_ uptake during the last minute of the test was calculated and reported as VO_2peak_.

Additionally, participants in the Exercise group performed a graded exercise test to task failure (cadence <60 rpm) under normobaric hypoxic condition (F_I_O_2_ = 0.14) to determine the altitude specific peak power output that was used to calculate exercise training intensity. During the test, the participants breathed the humidified hypoxic gas mixture through a facemask, using a low resistance two-way valve (2700 NRBV, Hans Rudolph Inc., Shawnee, U.S.).

#### Exercise training

The exercise group participants performed two 60-min training sessions daily on a bicycle fitted to a mechanically braked indoor cycle trainer (Elite SuperChrono Forte, Fontaniva, Italy). Exercise sessions were performed during the morning and afternoon hours under the same environmental conditions (F_I_O_2_  = 0.140±0.004; P_I_O_2_  = 88.8±0.6 mmHg). The targeted training HR was 140±8 beats·min^−1^ corresponding to the HR measured at 50% of the altitude specific peak power output attained during the baseline hypoxic test. HR and SpO_2_ exercise responses were recorded using a short-range telemetry system (iBody, Wahoo Fitness, Atlanta, U.S.) and a finger oximetery device (3100 WristOx, Nonin Medicals, Minnesota, U.S.), respectively. The Sedentary group participants were not allowed to perform any static or dynamic exercise throughout the confinement period.

#### Body mass and composition

Body mass, whole-body composition and regional body composition and (% body fat, fat mass and fat-free mass (FFM)) were assessed at Pre and Post using a fan-beam dual-energy X-ray absorptiometry scanner ((DXA) Discovery W - QDR series, Hologic, Bedford U.S.). To optimize the reproducibility of the DXA measures the participants were always scanned in the morning, fasted and well rested. Both leg and android (abdomen) regions of interest used for the regional analysis were defined as detailed previously [32]. The precision of DXA for *in vivo* body composition measurements has previously been demonstrated [33]. The scans were subsequently analyzed by the same experienced researcher using the Hologic APEX System Software (version 3.1.2).

#### Meal tolerance test (MTT)

The MTT protocol is outlined in Figure 1. The MTT was performed in a quiet, thermally comfortable laboratory (temperature  = 22.6±0.9°C; relative humidity  = 48.3±6.8%), with participants in a semi-supine position. The first MTT was performed in the morning of the Day -1 (Pre) and the second commenced following exactly 240-hrs of confinement on the morning of Day +1, prior to exiting hypoxic environment (Post). The participants refrained from exhaustive exercise, and from alcohol and coffee consumption and were placed on a standardized, prescribed diet for two days prior to the first MTT. The last meal before both tests was a standardized dinner served exactly 12-hrs before the start of the MTT. The MTT comprised fasting sample collection, standardized meal ingestion at time 0, and subsequent postprandial sample collection for two hrs. Breath samples for determination of ^13^C isotope in expired CO_2_, used as an indicator of the glucose uptake and the rate of gastric emptying, were obtained at 15-min intervals throughout the MTT. Perceived appetite ratings were obtained at 30-min intervals during the MTT. A standardized liquid meal (Ensure Plus vanilla flavor, Abbott nutrition laboratories Ltd., Maidenhead, U.K.) with an energy value of 1.5 kcal·mL^−1^ was used. Both, the meal volume (5 mL·kg^−1^) and the amount of diluted ^13^C isotope-labeled glucose (9.2 mg·kg^−1^) were calculated according to each individual's body weight on the day of the MTT. The average volume and energy content of the liquid meal were 366.8±40.5 mL and 550±61 kcal, respectively. The average liquid meal comprised 73.9±8.1 g of carbohydrate, 22.8±2.5 g of protein and 18.0±1.9 g of fat. The participants were requested to consume the liquid meal in its entirety.

#### Blood sampling

Arterialized venous blood samples were obtained from a 20-gauge cannula (Venflon, Becton Dickinson Infusion Therapy, Helsingborg, Sweden) inserted retrograde into a vein on the dorsum of the wrist or the forearm. A 7-cm extension catheter (Connecta, Becton Dickinson Infusion Therapy, Helsingborg, Sweden) was attached to allow blood to be sampled away from the hand. The catheterized arm was placed in a hot box (Handwarmer, MEU University of Nottingham, Nottingham, U.K.) at least 15 minutes before the first blood sampling to provide a constantly high hand temperature throughout the MTT (air temperature within the heated box ∼55°C). The cannula was flushed with saline solution (0.9% Sodium Chloride, Baxter Healthcare, Norfolk U.K.) after each sampling. To account for the cannula dead space and saline blood dilution, two mL of blood was drawn and discarded before each sampling. The first two fasting samples (8 mL) were obtained before the ingestion of the meal (−15 & −5 min) with additional samples (6 mL) collected at 15-min intervals throughout the MTT (15, 30, 45, 60, 75, 90, 105 & 120 min). The plasma concentration of total ghrelin, PYY, GLP-1, insulin and plasma glucose were determined at all sampling points, while the concentrations of serum leptin, adiponectin, high-density lipoprotein cholesterol (HDL-C), low-density lipoprotein cholesterol (LDL-C), total cholesterol, plasma free-fatty acids (FFA), triacylglycerols (TAG), and catecholamines (adrenaline & noradrenaline) were only determined from the two fasting samples. A 0.5 mL blood sample was also obtained every 10 min to monitor the plasma glucose concentration throughout the MTT. To determine any acute changes in the measured markers an additional fasting venous blood sample (6 mL) was obtained following the first 24-hrs of confinement (morning of Day 2), by venepuncture from the antecubital vein.

The sampled blood for determination of total ghrelin and PYY was collected into a precooled EDTA tube (Vacutainer K2E, Becton Dickinson, Plymouth, U.K.) containing 50 µl of Trasylol, whereas a pre-cooled EDTA tube containing 50 µl of Dipeptidyl peptidase-4 was used for GLP-1 determination. For fasting TAG and catecholamine assessment, the blood was collected into a continuously ice-cooled Lithium Heparin tube (Vacutainer LH, Becton Dickinson, Plymouth, U.K.) containing 50 µl of ethylene glycol tetraacetic acid (EGTA) and 10 µl of the lipase inhibitor, tetrahydrolipostatin. 50 µl of EGTA was added to a precooled Lithium Heparin tube (Vacutainer LH, Becton Dickinson, Plymouth, U.K.) used to collect the fasting sample for FFA determination. These samples were immediately centrifuged (3500 rpm; 10-min @ 4°C) using a laboratory centrifuge (Centric 200R, Tehtnica Železniki, Železniki, Slovenia). The remaining sample was collected into a separate tube (Vacutainer SST II Advance tube, Becton Dickinson, Plymouth, U.K.) for serum insulin, leptin, adiponectin and cholesterol determination, and centrifuged, as detailed above, after standing at room temperature for 15 minutes to allow clotting to occur. Blood samples for glucose concentration determination were collected in a safe-seal fluoride oxalate tube (Microtube FH, Sarstedt, Nümbrecht, Deutschland) and immediately centrifuged (13500 rpm; 1-min @ ∼22°C) in a micro centrifuge (Mini, Labogene, Lynge, Denmark). The plasma was transferred into a microtube (Microtube Cap, Sarstedt, Nümbrecht, Deutschland) and frozen at −80°C immediately after the MTT for subsequent analysis performed less than 6 months after the study.

#### Biochemistry

The analysis of all plasma and serum samples was performed in duplicate using the methods outlined below. Appropriate radioimmunoassays (EMD Millipore, Billerica, U.S.) were used to determine the concentration of total ghrelin, PYY, insulin, leptin, and adiponectin. GLP-1 concentration was determined using sandwich ELISA (EMD Millipore, Billerica, U.S.), comprising amide acid GLP-1 7-36 and 7-37. The glucose concentration was assessed using the glucose oxidase enzymatic method (YSI Inc., Yellow Springs, Ohio, USA).

The concentration of FFA, TAG, HDL-C, LDL-C and total cholesterol was determined using spectrophotometry (ABX Pentra 400, Horiba Medical, Montpellier, France). Adrenaline and noradrenaline concentrations were determined by extraction of both from the plasma (HPLC – ECD; Glutathione preservative E4378, Sigma-Aldrich Company Ltd., Dosert, U.K.), followed by high performance liquid chromatography with electrochemical detection [34]. The intra-assay coefficient of variation (CV) for leptin, FFA, TAG, HDL-C, LDL-C and total cholesterol (analyzed in a single run) were 1.0%, 1.5%, 2.9%, 1.3%, 1.0% and 4.6%, respectively. The inter-assay CV for total ghrelin, PYY, GLP-1, insulin, plasma glucose, adiponectin, adrenaline and noradrenaline (analyzed in multiple runs) were 9.1%, 6.7%, 4.9%, 7.4%, 2.3%, 8.6%, 11.8% and 17.2%, respectively.

#### Isotope ^13^C tracer protocol

Ubiquitously labeled ^13^C glucose, tested for pyrogenicity and sterility (Cambridge Isotope Laboratories, Inc., Andover, U.S.) was dissolved in the individual's liquid meal immediately prior to the MTT. The breath sampling protocol is based on the principle that the ingested substrate is absorbed, metabolized, and a measurable metabolite subsequently expelled through the respiratory system. The rate of appearance of ^13^C isotope in exhaled breath CO_2_ is a product of the rate of gastric emptying, absorption from the gut, uptake by metabolizing tissues and oxidation of the liquid meal. Exhaled breath samples were collected into a 500 mL breath-sampling bag using a one-way valve, before the meal ingestion (−15 & −5 min) and at 15-min intervals postprandially (15, 30, 45, 60, 75, 90, 105 & 120 min). At each sampling point, duplicate 12-mL breath vials (Exetainer, Labco Limited, Lampeter, U.K.) were filled from the breath-sampling bag using a catheter (Vacutainer adapter, Becton Dickinson, Plymouth, U.K.). All ^13^CO_2_ samples were analyzed in triplicate using continuous flow isotope ratio mass spectrometry (VG Isochrom, VG Analytical, Manchester, U.K.) less than six months after the study.

#### Perceived appetite ratings

Visual analogue scores (VAS) were obtained before and during the MTT using a custom-designed application on an iPad tablet (Apple, Cupertino, USA) to assess perception of hunger, fullness, desire to eat, and prospective food consumption (PFC) [35]. The VAS scale was a 100 mm digital line, anchored to the left with ‘sensation not felt at all’ and to the right with ‘sensation felt the greatest’. A similar VAS collection method using a handheld electronic device has been validated previously [36]. The participants were instructed to place a vertical line according to their perceived feeling at that particular point in time for all included questions. VAS were filled by the participants 5 min before the liquid meal ingestion and 30 min, 60 min, 90 min and 120 min thereafter. The Composite Satiety Score (CSS) was subsequently calculated from the obtained scores using the following formula (higher the score, higher the level of subjective satiety): CSS  =  (Fullness + (100 − Desire) + (100 − Hunger) + (100 – PFC))/4. CSS scores were also obtained throughout the confinement period before and after breakfast, lunch and dinner.

#### Statistical analysis and calculations

All data were coded and analyzed using Statistica 12.0 for Windows (StatSoft, Tulsa, USA). Prior to analysis the data were tested for normality of distribution using a modified Kolmogorov-Smirnov test. Student's independent-samples t-test was used to compare participants' baseline characteristics and average daily energy and macronutrient intakes between groups. Area under the curve (AUC) was calculated for total ghrelin, PYY, GLP-1, insulin, plasma glucose and CSS changes during the MTT, using the conventional trapezoid rule. Due to blood clotting and resulting inability to obtain the last two blood samples during one of the Post MTT tests the AUC values were calculated over 90-min for total ghrelin, PYY, GLP-1 and insulin and over 100-min for glucose. The Pre data obtained at 105 and 120-min from this individual were omitted from the analysis. A 1-way repeated measures unbalanced ANOVA analysis was used to define the effect of the 10-day confinement on BP, HR, SpO_2_, LLS [group (Exercise & Sedentary) × Time (Day1–Day10)] and fasting metabolic markers (leptin, adiponectin, FFA, TAG, cholesterol and catecholamines), calculated AUC values and body-composition changes [group (Exercise & Sedentary) × testing period (Pre & Post)]. A 2-way repeated measures unbalanced ANOVA [group (Exercise & Sedentary) × testing period (Pre & Post) × time (MTT Pre & Post prandial responses)] was used to define the effect of the 10-day confinement on the CSS and metabolic markers, measured throughout the MTT (total ghrelin, PYY, GLP-1, insulin, glucose). Tukey's HSD *post hoc* test was employed to define the specific differences when ANOVA analysis revealed a significant F-ratio for the main effect or an interaction. Pearson's correlation coefficient was used to define bivariate correlations between changes in whole body fat and fat free mass (Pre-Post) and changes in fasting levels of total ghrelin, PYY, GLP-1, insulin, glucose and leptin. Values are represented as mean ± SD in the text and tables and as mean ± SEM in the figures. Statistical significance was set a priori at α = 0.05.

The ^13^C glucose results are expressed as a change of ^13^C using the ^13^CO_2_ enrichment equations. The differences between the measured ^13^C enrichment in the expired CO_2_ and the international standard for ^13^C abundance (0.010767) were calculated and expressed as atoms %. The values were normalized and define the total amount of units for delta change after a standardized meal (total amount of the substrate metabolized). Insulin resistance was quantified before (Pre) and after (Post) the confinement by the homeostatic model assessment (HOMA IR) method [37] using the following formula: HOMA IR  =  (fasting insulin (mU·l^−1^)) × (fasting glucose (mmol·l^−1^))/22.5.

For metabolic parameters with significant main effects or interactions, ANOVA revealed the following observed statistical analysis power: 0.98 for total ghrelin (time effect), 0.96 for PYY (time effect), 0.98 for GLP-1 (time effect), 0.87 for insulin (time × testing period effect), 0.78 for glucose (time × testing period effect), 0.93 for leptin (time effect), 0.89 for adiponectin (time effect), 0.64 for TAG (time effect), 0.82 for noradrenalin (time effect), 0.67 for LDL-C (time × group effect), 0.85 for total cholesterol (time effect) and 0.84 for CSS (time effect).

## Results

### Participants

Except for one participant with acute appendicitis, and one with general adaptation problems, all others underwent the confinement without any significant adverse effects. AMS was present in 4 participants in the Exercise group, and 3 in the Sedentary group on Day 1. The average LLS values were comparable between groups throughout the confinement (Exercise  =  1.5 [0–6]; Sedentary  =  1.4 [0–7]; mean [range]).

The participants in the Exercise group performed all 20 training sessions. The average training HR responses (142±11 beats·min^−1^) were within the targeted levels and the average SpO_2_ values were 85% [76–89%] (mean [range]). Average daily systolic, but not diastolic BP was higher in the Exercise group than in the Sedentary group (125±9 mmHg vs 119±12 mmHg; p = 0.01). A significant decrease in resting daily HR over the confinement period was only noted in the Exercise group (p<0.01), with HR decreasing from 100±12 beats·min^−1^ at Day 1 to 80±12 beats·min^−1^ on Day 10. The SpO_2_ was higher on Day 10 (87 [83–92%]) than on Day 1 (81 [77–84%]; p<0.01) in the Exercise group, and tended to be lower compared to Sedentary group on Day 3 (Exercise  =  83 [78–88%]; Sedentary  =  88 [86–91%]; p = 0.09 (mean [range])).

### Diet and fluid intake

The average daily energy, macronutrient, water, and selected micronutrient intakes of both groups are presented in Table 1. Compared to the pre-calculated levels the actual average energy intake was significantly lower only in the Exercise group (2601±383 kcal; p<0.01), while the Sedentary group intake only tended to be lower (2213±192 kcal; p>0.05). Average daily total water intake, was significantly higher in the Exercise (4.87±1.46 L; p<0.01) compared to the Sedentary (3.28±1.34 L) group. As a consequence of greater food consumption, the iron, sodium, and calcium intakes were significantly higher in the Exercise compared to the Sedentary group (p<0.05) (Table 1).

### Body mass and composition

Body mass, whole body fat mass, whole body FFM as well as leg FFM were significantly lower in the Sedentary than the Exercise group prior to the study. Body mass was significantly reduced in both groups following the 10-day confinement period (Exercise  =  −1.4 kg; Sedentary  =  −1.4 kg; p<0.05). As noted in Table 2, whole body fat mass was significantly reduced after the confinement in the Exercise group whereas no changes were observed in whole body % body fat and FFM (p>0.05) in either group. After the confinement, the leg fat mass significantly decreased (p = 0.03) and the leg FFM significantly increased (p = 0.01) in the Exercise group only. No differences significant differences were observed in the regional android fat or FFM (Table 2).

**Table 2:** Body mass, whole body (WB) composition and regional leg and android (abdomen) composition analysis (DXA) in both Exercise and Sedentary group before (Pre) and after (Post) the 10-Day normobaric hypoxic confinement.

	Exercise group		Sedentary group	
	Pre	Post	Pre	Post
Body mass (kg)	75.2±6.4	73.8±6.1#	68.8±10.1**	67.4±9.8#**
WB fat mass (kg)	17.4±5.9	15.9±5.4##	15.6±4.8**	14.7±5.1
WB fat free mass (kg)	57.8±3.9	57.9±3.7	53.2±5.4**	52.7±4.9
% Body fat (%)	22.8±6.2	21.2±5.8	21.6±4.4	21.3±4.8
Leg fat mass (kg)	6.3±0.6	5.6±0.6#	5.9±0.5	5.6±0.5
Leg fat free mass (kg)	17.5±0.5	18.0±0.4##	16.4±0.6**	16.1±0.4
Android fat mass (kg)	1.2±0.4	1.1±0.4	1.1±0.5	1.1±0.4
Android fat free mass (kg)	3.8±0.3	4.0±0.3	3.7±0.5	3.6±0.5

Values are means ± SD. ** indicates significant differences compared to the Exercise group (p<0.01);

# indicates significant differences compared to Pre (# p<0.05, ## p<0.01).

### Hormonal appetite markers

**Figure 2:**
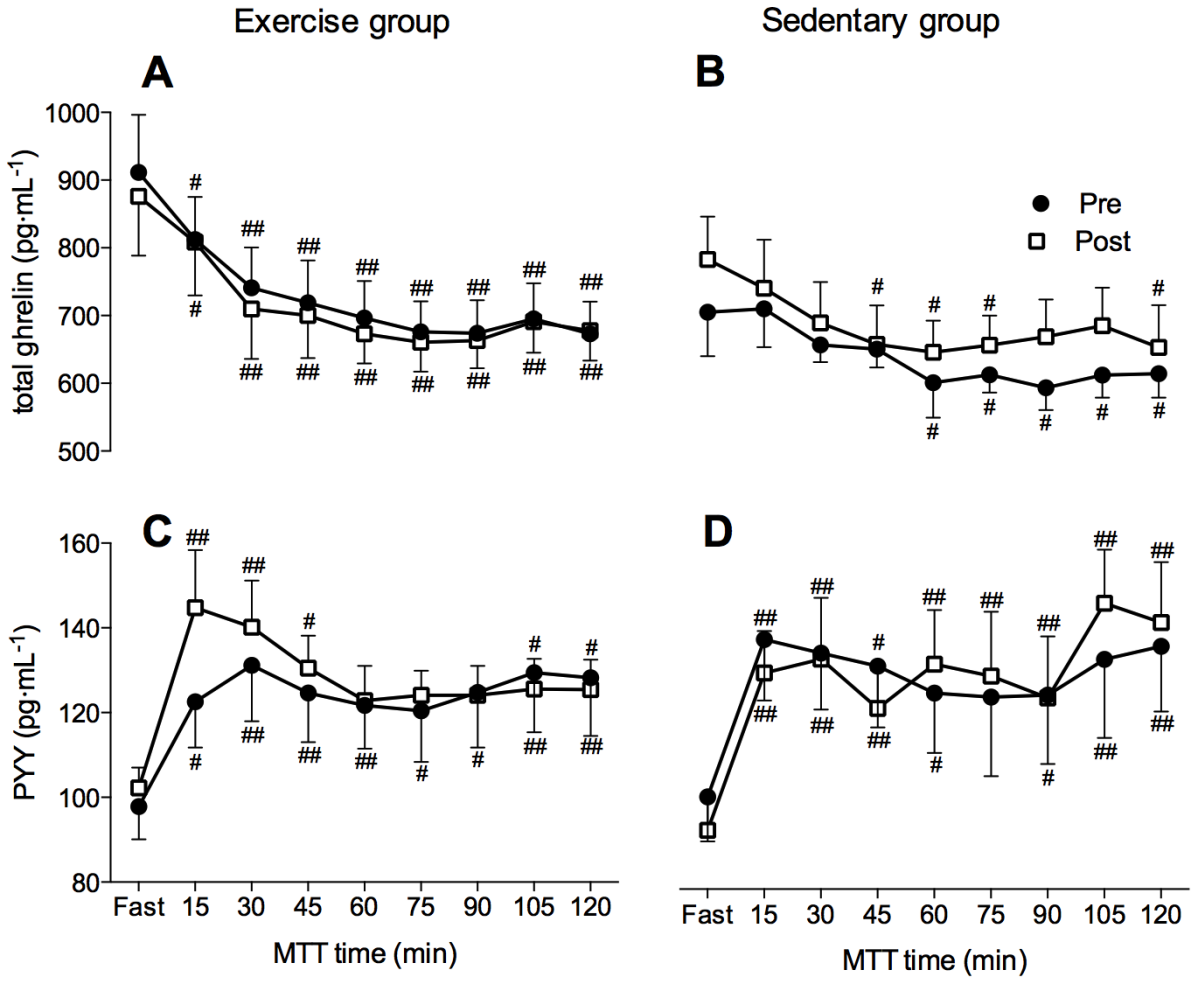
Total ghrelin and peptide YY (PYY) kinetics during the meal tolerance test in both Exercise (A, C) and Sedentary (B, D) group, respectively. The meal tolerance test (MTT) was performed before (Pre; closed circles) and after (Post; open squares) the confinement. Two-way repeated measures ANOVA analysis, followed by a Tukey's HSD *post hoc* test; # indicates significant differences compared to fasting values (# p<0.05; ## p<0.01). Values are mean ± SEM.

**Figure 3:**
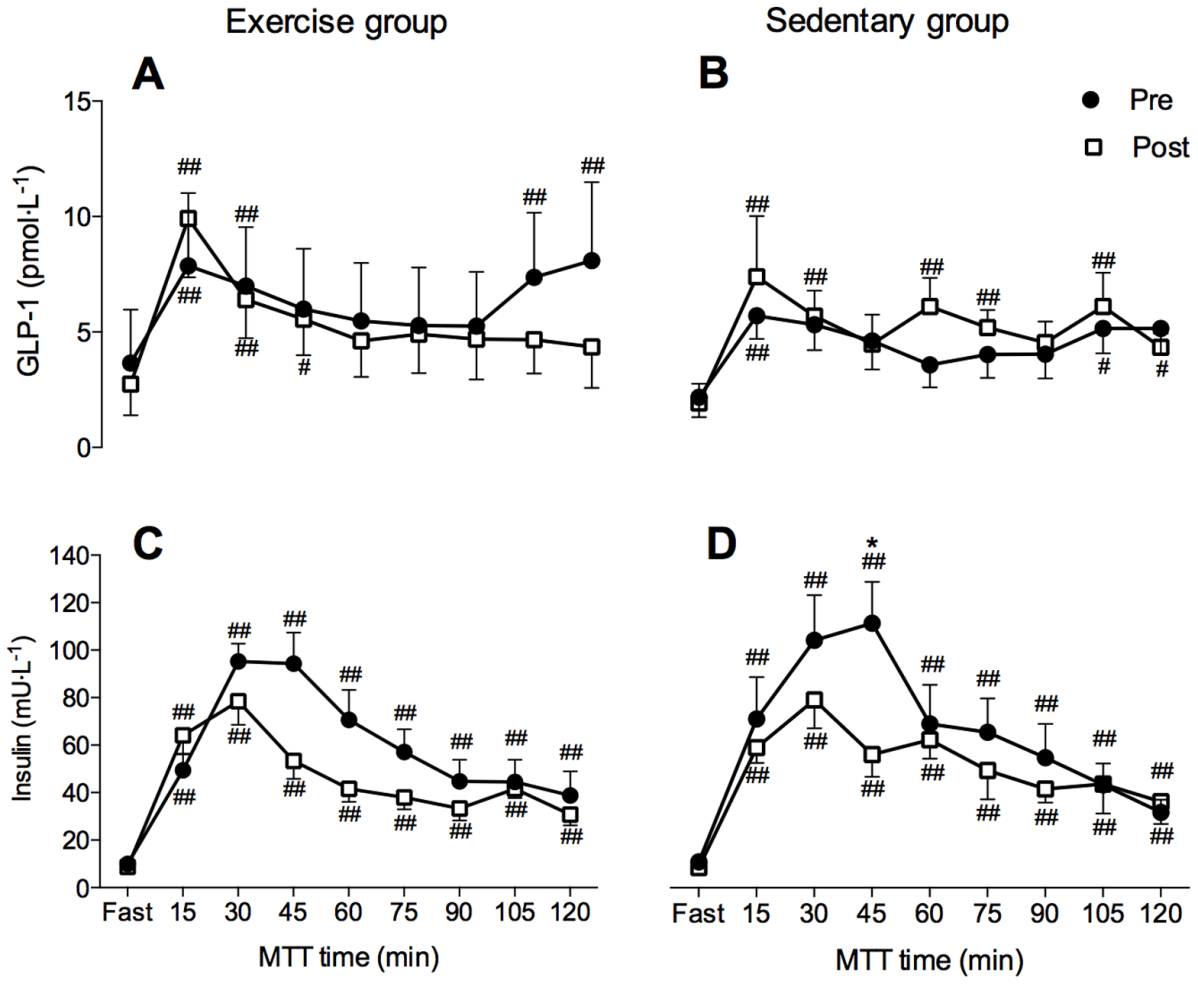
Glucagon-like peptide-1 (GLP-1) and insulin kinetics during the meal tolerance test in Exercise (A, C) and Sedentary (B, D) group, respectively. The meal tolerance test (MTT) was performed before (Pre; closed circles) and after (Post; open squares) the confinement. Two-way repeated measures ANOVA analysis, followed by a Tukey's HSD *post hoc* test; # indicates significant differences compared to fasting values (# p<0.05; ## p<0.01). * indicates significant differences compared to Pre values (p<0.05). Values are mean ± SEM.

Fasting levels and postprandial levels of total ghrelin, PYY and GLP-1 were not significantly different between groups (p>0.05), or between Pre and Post MTT tests within groups (p>0.05; Figure 2, 3). As noted in Table 3, fasting morning values of total ghrelin, PYY and GLP-1 were similar on Day 2 compared to Pre (p>0.05). The AUC values for total ghrelin, PYY and GLP-1 was also comparable between groups at Pre and did not significantly change at Post (p>0.05; Table 4). No significant differences were noted in fasting leptin and adiponectin levels (p>0.05) between Pre and Post values and between the groups (Table 3).

**Table 3:** Fasting levels of total ghrelin, peptide YY (PYY), glucagon-like peptide-1 (GLP-1), insulin, leptin, adiponectin, free fatty acids (FFA), triacylglycerols (TAG), adrenaline and noradrenaline in both Exercise and Sedentary group before (Pre), following the first 24-hrs of confinement (Day 2) and immediately after the 10-day confinement (Post).

	Exercise group			Sedentary group		
	Pre	Day 2	Post	Pre	Day 2	Post
Total ghrelin (pg·mL^−1^)	911±239	817±162	876±247	705±160	743±145	782±155
PYY (pg·mL^−1^)	97.8±21.8	93.8±13.3	102.2±13.6	101.7±25.6	96.8±31.2	93.8±20.7
GLP-1 (pmol·L^−1^)	3.65±6.6	3.75±5.84	2.75±3.82	1.92±2.12	2.62±2.35	1.67±2.04
Insulin (mU·L^−1^)	10.6±2.0	10.9±1.9	8.7±1.9	10.4±1.5	12.1±1.9	8.1±1.1##
Leptin (µg·L^−1^)	4.91±3.53	6.92±4.22	3.65±2.02##	3.60±1.22	5.03±2.14	3.49±1.36
Adiponectin (µg·mL^−1^)	7.41±3.26	7.97±3.63	6.12±3.43#	8.03±2.23	8.09±1.68	7.05±0.51
FFA (mmol·L^−1^)	0.44±0.23	0.56±0.24	0.44±0.15	0.52±0.22	0.45±0.20	0.52±0.14
TAG (mmol·L^−1^)	0.52±0.16	0.55±0.17	0.56±0.20	0.46±0.25	0.73±0.35*	0.55±0.28
Adrenaline (nmol·L^−1^)	0.27±0.05	0.24±0.14	0.23±0.06	0.19±0.08	0.19±0.13	0.24±0.07
Noradrenaline (nmol·L^−1^)	0.86±0.35	1.52±0.51*	1.16±0.26	0.95±0.67	1.29±0.49	1.28±0.39

Values are means ± SD. * indicates significant differences compared to Pre (p<0.05); # indicates significant differences compared to Day 2 (# p<0.05, ## p<0.01).

**Table 4:** Area under the curve calculated from total ghrelin, peptide YY (PYY), Glucagon-like peptide-1 (GLP-1), insulin, glucose and Composite satiety score (CSS) kinetics in both Exercise and Sedentary group during the meal tolerance test performed before (Pre) and after (Post) the 10-Day normobaric hypoxic confinement.

	Exercise group		Sedentary group	
	Pre	Post	Pre	Post
Total ghrelin (pg·mL^−1^·90 min^−1^)	44379±9721	43220±10043	37808±5582	41157±8030
PYY (pg·mL^−1^·90 min^−1^)	7318±1854	7755±1416	7714±2119	7597±1767
GLP^−^1 (pmol·L^−^1·90 min^−1^)	360±437	351±285	247±136	305±159
Insulin (mU·L^−1^·90 min^−1^)	3941±1138	2965±561##	4348±2006	3170±849##
Glucose (mmol·L^−1^·100 min^−1^)	664±63	605±44	662±78	631±59
CSS (mm·120 min^−1^)	2061±286	2391±288	2240±172	2092±154

Values are means ± SD. ## indicates significant differences compared to Pre (p<0.01).

Changes (Pre-Post) in fasting leptin concentration were significantly correlated to changes in whole body fat mass following the confinement (Pre-Post; r = −0.70, p = 0.01). No correlation was found between changes in whole body fat mass and whole body FFM and changes in fasting levels of total ghrelin (r = 0.33, r = 0.02, p>0.05), PYY (r = −0.32, r = 0.38, p>0.05), GLP-1 (r = 0.05, r = −0.01, p>0.05).

### Perceived appetite ratings

**Figure 4:**
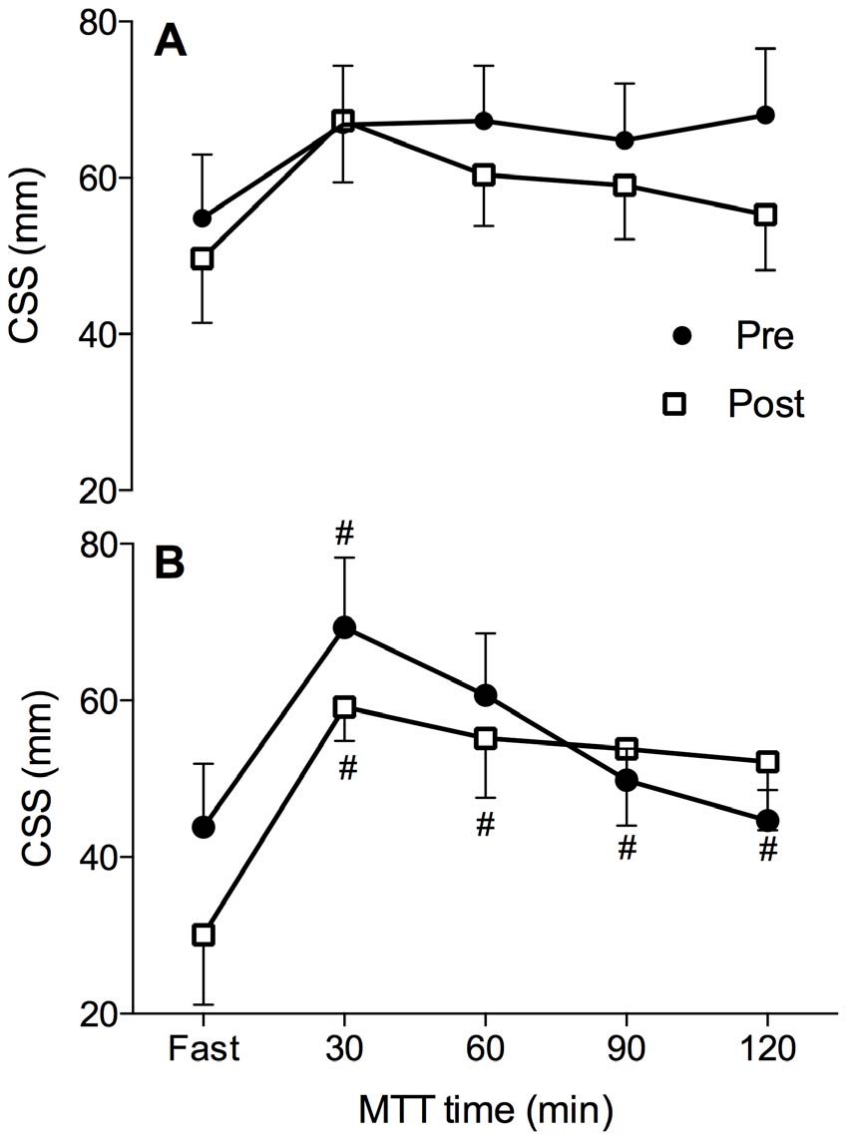
Composite satiety scores before (Fast) and at 30-min intervals throughout the meal tolerance test for both Exercise (A) and Sedentary group (B). One-way repeated measures ANOVA followed by Tukey's HSD *post hoc* test; # indicates significant differences compared to fasting values (p<0.05). Values are mean ± SEM.

The CSS values during the MTT are presented in Figure 4. No significant differences were noted either between groups (p>0.05), or between Pre and Post testing (p>0.05). No significant differences in CSS were observed throughout the MTT in the Exercise group (p>0.05). Also, CSS AUC was not significantly different between groups and testing periods (p>0.05; Table 4). Morning fasting daily CSS values were comparable between groups throughout the whole confinement period except on Day 2 with significantly lower values in the Exercise (44±6 mm), than the Sedentary group (56±7 mm; p<0.05) as well as compared to Pre fasting values (53±5 mm; p<0.05). The average aggregate daily CSS values obtained throughout the confinement period were comparable between groups (Exercise group  =  63±2 mm, Sedentary group  = 62±3 mm; p>0.05)

### Insulin sensitivity

Insulin resistance, quantified by HOMA IR was similar between groups at Pre and only significantly decreased following the confinement in the Sedentary group (Exercise group: Pre  =  2.23±0.47, Post  = 1.85±0.43, p>0.05; Sedentary group: Pre  = 2.27±0.38; Post  = 1.71±0.25, p = 0.03).

**Figure 5:**
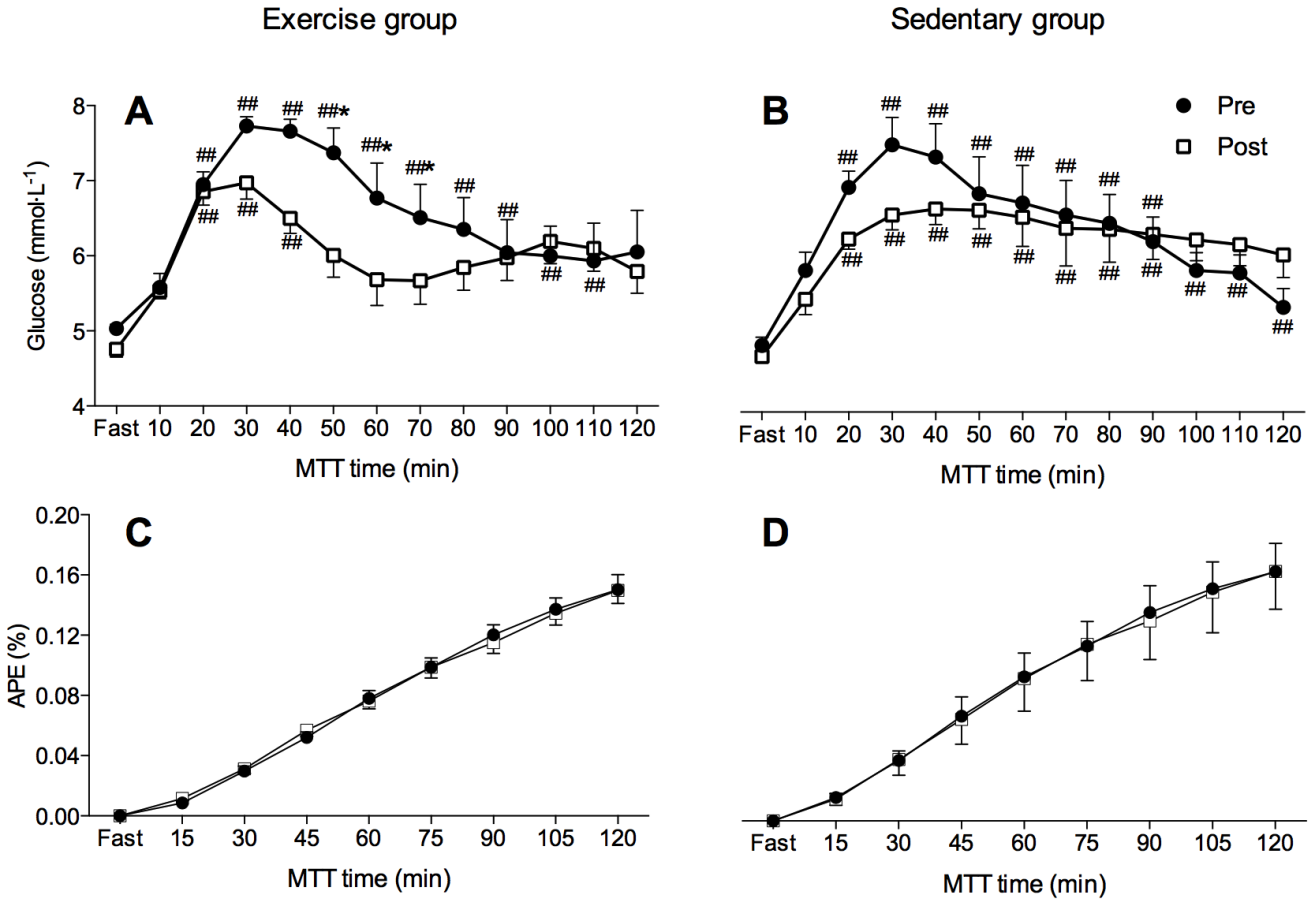
Plasma glucose kinetics and atom percent excess (APE) of expired CO_2_ isotopic enrichment changes during the meal tolerance test in Exercise (A, C) and Sedentary group (B, D), respectively. The meal tolerance test (MTT) was performed before (Pre; closed circles) and after (Post; open squares) the confinement. Two-way repeated measures ANOVA followed by Tukey's HSD *post hoc* test; ## indicates significant differences compared to fasting values (p<0.01). * indicates significant differences compared to Pre values (p<0.05). Values are mean ± SEM.

No differences were noted between groups in fasting and postprandial insulin kinetics (p>0.05) at both study days. Compared to Pre, the insulin levels were significantly reduced at 40-min postprandial sampling during the Post MTT (p = 0.01; Figure 3) in the Sedentary group. The insulin AUC was significantly reduced in both groups following the confinement (p = 0.04; Table 4). Fasting glucose was comparable between groups and did not change following the confinement (Exercise group: Pre  =  5.03±0.16, Post  = 4.76±0.30; Sedentary group: Pre  = 4.89±0.27; Post  = 4.74±0.18; p>0.05). Postprandial glucose concentration was significantly lower at 50–70 min sampling at Post compared to Pre in the Exercise group only (p = 0.03; Figure 5). Postprandial glucose was significantly increased between 20–90 min sampling points during the Pre (p = 0.04) and at 20–40 min and 100–110 min samplings during the Post (p = 0.01) in the Exercise group (Figure 5). In the Sedentary group, glucose was elevated postprandially between 20–90 min sampling during the Pre and between 20–120 min during the Post MTT (p = 0.01). A trend for a reduction in glucose AUC following the confinement was observed in the Exercise group only (p = 0.07; Table 4).

### Isotope ^13^C

As illustrated in Figure 5, no significant differences (p>0.05) were observed between the Exercise and Sedentary group or between Pre and Post testing periods in atom percent excess of expired CO_2_ isotopic enrichment kinetics following the standardized meal ingestion.

### Lipid profile and catecholamines

**Figure 6:**
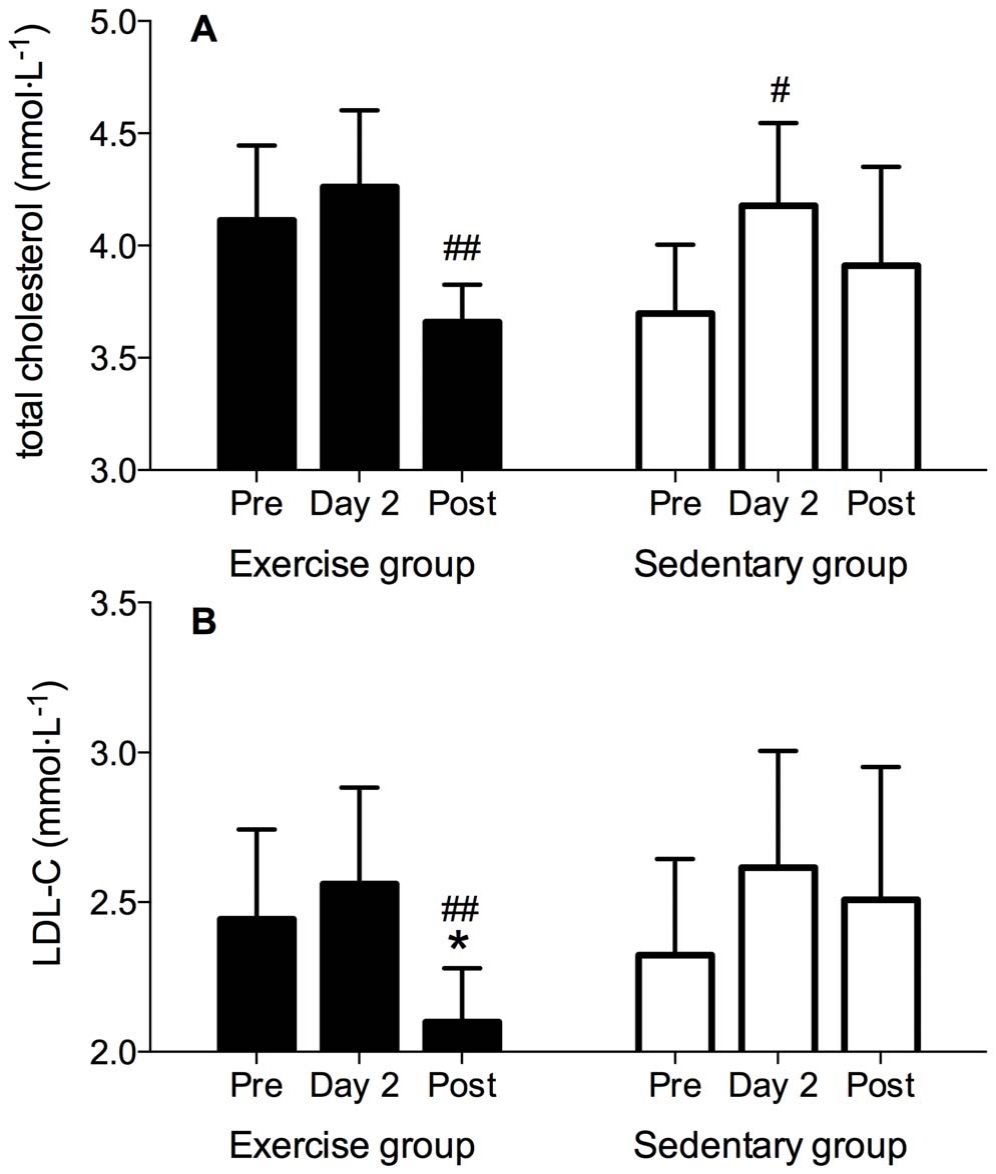
Fasting total cholesterol (A) and low-density lipoprotein cholesterol (LDL-C) levels in both Exercise and Sedentary group before (Pre), following the first 24-hrs of confinement (Day 2) and immediately after the confinement (Post). One-way repeated measures ANOVA followed by Tukey's HSD *post hoc* test; # indicates significant differences compared to Pre (# p<0.05; ## p<0.01). * indicates significant differences compared to the Sedentary group (p<0.05). Values are mean ± SEM.

Compared to Pre the TAG concentration was significantly increased in the Sedentary group (p = 0.03) on Day 2 (Table 3). No significant differences were observed in fasting levels of FFA (p>0.05) and TAG (p>0.05) between Pre and Post values and between the groups (Table 3). Total cholesterol was significantly increased in the Sedentary group on Day 2 (p = 0.02) and reduced at Post in the Exercise group only (p = 0.01; Figure 6). As noted in Figure 6, LDL-C was significantly reduced in the Exercise group at Post (p = 0.01). The LDL-C values at Post were lower in the Exercise compared to the Sedentary group (p = 0.04). No differences were observed in the Sedentary group or in HDL-C values between groups or testing periods (p>0.05).

Noradrenaline levels were significantly increased on Day 2 in the Exercise group only (p = 0.01) (Table 3). No significant differences were observed in fasting adrenaline (p>0.05) or noradrenaline (p>0.05) levels between Pre and Post values or between the two groups (Table 3).

## Discussion

### Main findings

This study investigated the effects of prolonged hypoxic exposure combined with, or without exercise training on hormonal appetite regulation and select metabolic markers. Contrary to our initial hypothesis, adding exercise to a 10-day hypoxic confinement did not significantly affect hormonal appetite regulation in fasted and postprandial states. Similarly, based on the ^13^C tracer results, the passage of glucose from ingestion to breakdown and CO_2_ excretion was also unaffected. The potentially beneficial effects of adding exercise to hypoxia observed in this study were the reduction in postprandial glucose response, a significant decrease in fasting total cholesterol, secondary to LDL-C reduction and a reduction of whole body fat mass.

### Hypoxia and hormonal appetite regulation

Ghrelin is the only known appetite stimulating gut peptide, and it has been suggested to play a role in the altitude-related appetite modulation. Acute [6]–[8], but not chronic [7] hypoxic exposure has been shown to reduce circulating ghrelin levels. Interestingly, the fasting ghrelin levels in our study were unchanged both upon acute exposure (24-hrs), and after the 10-day exposure to a simulated altitude of 4000 m. Furthermore, postprandial ghrelin kinetics and the ghrelin AUC were also unaffected by hypoxic confinement *per se*. As the degree of hypoxia (∼3500–4200 m altitude) and AMS responses were similar in the present and aforementioned studies [6]–[8], factors underlying the different outcomes are not obvious. These might include the diet and mode of hypoxia (i.e normobaric vs. hypobaric). It has to be noted however, that the lack of change in ghrelin concentration was also previously observed following chronic high-altitude altitude exposure [9].

The measured anorexigenic modulators leptin, GLP-1 and PYY were also unaffected by prolonged hypoxic exposure in our study. This seems contrary to the majority of other studies indicating that the levels of the satiety-inducing adipokine leptin increase as a result of acute hypoxic exposure [7], [10], [11]. However, the response of leptin to hypoxia has been widely debated and collective results from the reported studies are equivocal. Leptin has also been related to GLP-1 production in a rodent model, and subsequently a leptin appetite suppression through a GLP-1 mediated pathway was proposed [38]. To date only one study examined the response of GLP-1 to acute hypoxia and showed a tendency for increased GLP-1 postprandially with no changes in the fasting state [10]. The dependent relation between leptin and GLP-1 could therefore play a role in the lack of appetite regulation changes observed in this study. Similarly to GLP-1, only one study to date examined the effects of hypoxic exposure on PYY concentration and observed a tendency for lower PYY values in response to acute hypoxia than to normoxia [8]. Thus, implying that PYY might not be involved in hypoxia-induced appetite modulation. Our data also lend support to this notion as no significant changes in PYY after acute or prolonged hypoxic exposure were observed. The lack of changes in the measured hormonal appetite regulation markers following hypoxic confinement without concomitant exercise is also corroborated by the unchanged fasting and postprandial ratings of perceived appetite throughout the 10-day confinement. Collectively, the responses of the Sedentary group participants indicate that acute or prolonged exposure to simulated altitude of 4000 m does not seem to alter fasting and postprandial (2-hrs) concentrations of ghrelin, leptin, GLP-1 and PYY plasma.

### Hypoxia, exercise and hormonal appetite regulation

While many studies investigating concomitant hypoxia and exercise aimed to determine the potential beneficial effects on performance in athletes [19], very few scrutinized the combined effects on appetite regulation and metabolic health [8]. Moreover, no long-term study to date attempted to discern the separate effects of exercise, often associated with high altitude sojourns, and prolonged hypoxia on hormonal appetite regulation.

The hypothesis that combination of hypoxia and exercise might down-regulate appetite through modulation of orexigenic and anorexigenic hormones was recently tested in healthy sedentary men [26]. The participants in this study performed 12 training sessions over a four-week period (3 training sessions per week) under either hypoxia (∼4000 m) or normoxia. Similarly to the current study they did not find any changes in fasting ghrelin, leptin and GLP-1 following hypoxic training although postprandial levels of leptin were increased in both the hypoxic and normoxic group. Interestingly, GLP-1 levels were only increased after the normoxic training. This is in line with previous studies demonstrating elevated GLP-1 levels following exercise training in normoxia [39], and suggests that GLP-1 might not play a role in combined exercise and hypoxia related appetite reduction. Wasse et al. [8] recently investigated ghrelin and PYY responses to acute hypoxic exposure, in combination with exercise and showed that there is a significant effect of both hypoxia and exercise on postprandial acylated ghrelin concentration. This was not confirmed in our study where total ghrelin was unchanged after the first 24-hrs of hypoxia as well as following 10 days of chronic hypoxia combined with exercise. This is in line with previous reports showing that ghrelin concentration can return to baseline upon prolonged exposure to hypoxia [7], [9]. However, it has to be noted that we also did not observe any acute changes in total ghrelin levels. No changes in PYY concentration after combined hypoxia and exercise were observed in this study, which support the notion that PYY might not play a role in hypoxia induced hormonal appetite regulation [8]. This notion is further reinforced by reports suggesting that the addition of hypoxia to exercise might even suppress PYY compared to identical exercise performed in normoxic conditions [40].

No change in the perceived appetite ratings was also noted in the Exercise group during or after the 10-day confinement in spite of the fact that the actual intakes were lower then targeted. Both observations together could be suggestive of a reduced motivation to eat during combined hypoxia and exercise training. Collectively, this corroborates the unchanged hormonal appetite measures and also, importantly, indicates that the physical activity factors used for dietary intake calculations (1.4 Sedentary; 1.8 Exercise) were appropriate for both conditions.

### Hypoxia, exercise and metabolic risk factors

Training in hypoxia has been shown to be superior to normoxic training for reducing fasting and postprandial insulin levels, insulin resistance [25] and improving glucose tolerance [41]. Based on these reports, and the fact that both hypoxia and exercise exert strong effects on the metabolic system, we reasoned that the addition of exercise to continuous hypoxic exposure would result in enhanced insulin sensitivity and lipid profiles. Especially, since hypoxia and exercise have been shown to up-regulate hypoxia-inducible factor 1α target genes [23] involved in glucose, oxygen uptake and lipid metabolism through increases in peroxisome proliferator activated receptor γ co-activator-1α [42]. However contrary to our hypothesis we did not observe any changes to measured metabolic variables as a result of exercise during prolonged hypoxia. Indeed the glycemic responses to feeding were similar in both groups, with significantly reduced insulin AUC at Post compared to Pre and no significant changes in glucose AUC in both groups. This finding supports the notion that hypoxia *per se* beneficially affects insulin postprandial kinetics [25], [41]. While the glucose AUC was unchanged following the confinement in the Sendentary group, a tendency for a decreased glucose AUC was observed in the Exercise group. This observation could be explained by the increased glucose turnover previously documented following prolonged acclimatization to high altitudes [43]. Taken together, the glucose AUC and significant postprandial glucose concentration reductions, suggest that performing exercise training during hypoxic confinement might also results in improved postprandial insulin sensitivity although this was not observed in the present study. This hypothesis is in line with recent studies showing that the use of hypoxia during exercise training additively improves insulin sensitivity in sedentary individuals [26] and glycemic control in individuals with type 2 diabetes [27].

It has to be noted however that the postprandial insulin levels were only significantly reduced following the confinement in the Sedentary group. No significant changes in postprandial insulin kinetics were observed in the Exercise group in spite of the fact that glucose was reduced. Furthermore, the postprandial values of both glucose and insulin were, albeit not significantly, lower at Post compared to Pre following the confinement in both groups. The fact that these reductions were predominantly not significant could be explained by the relatively small sample size, potentially limiting the statistical significance of the outcomes.

Although beneficial effects of combining hypoxia and exercise training on TAG have been reported previously [25], we did not observe any additional effect of exercise during continuous hypoxia. Interestingly, we noted a significant increase in TAG in the Sedentary group after the first 24-hrs of confinement. Moreover, we did not observe any significant changes in FFA concentration during and after the confinement in either group although FFA reductions were previously reported following exercise performed at moderate altitudes due to their increased metabolic utilization during exercise and recovery [24]. The substrate metabolism responses could also be altered by the exercise-induced changes in catecholamine levels. Indeed, hypoxic training has been shown to potentiate the exercise-induced sympathetic activation [44]. This is in line with our findings indicating a significant increase in noradrenaline after the first 24-hrs of hypoxia in the Exercise group only.

Another potentially beneficial metabolic response of exercise in the present study, besides a tendency for a reduced postprandial glucose profile was a significant reduction in LDL-C and a concomitant total cholesterol reduction. Athough this has previously been reported to occur following hypoxic training [45], other studies did not find any changes in either total cholesterol or LDL-C following combined hypoxia and exercise [25], [26].

In addition to select appetite and metabolic risk measures, the ^13^C glucose tracer protocol was implemented in this study to indirectly assess postprandial glucose uptake and related gastric emptying. Even though it has been speculated that changes in gastro-intestinal flow/permeability might be involved in “altitude anorexia” [46], no changes in postprandial expired CO_2_ isotopic enrichment kinetics were observed in this study. This is in line with other recent data [47] suggesting that both, glucose uptake and the rate of gastric emptying are unaffected by hypoxia.

### Methodological considerations

To our knowledge, this is the first controlled study that explored the effects of long-term continuous exposure to hypoxia in combination with exercise training on hormonal appetite regulation and selected metabolic responses. The hypoxic nature of our intervention is clearly demonstrated by significant reductions in SpO_2_ throughout the confinement period. While the SpO_2_ values gradually increased during the ten-day confinement, as a result of the well-established hypoxic ventilatory acclimatization, the average values did not exceed 89% at any time.

The main strengths of the present study are standardized diet (i.e. macronutrient diet composition) as well as general living conditions (i.e. sleep, activity, environmental conditions) throughout the 10-day confinement period. Other studies investigating prolonged effects of training in hypoxia did not precisely control both the dietary composition and intake levels and that might have significantly affected the metabolic measures [20], [21], [25], [26], [41]. It has to be noted that although the dietary intakes were matched between groups in terms of macronutrient intake percentages the intakes were significantly lower in the Sedentary than in the Exercise group. Moreover, the fact that the participants in the Exercise group did not attain the targeted intakes resulted in a negative energy balance, also documented by the reductions in whole body fat mass. We can only speculate whether the reductions in fat mass observed in the Exercise group were a result of hypoxia and exercise or a consequence of insufficient energy intakes. The observed reductions in whole body fat mass are in line with the study by Haufe et al. [25] reporting similar reductions following a four-week hypoxic exercise training intervention. Nevertheless, the observed energy deficit and body composition changes might have influenced our metabolic outcomes since a negative energy balance resulting from reduced dietary intake has previously been shown to influence hormonal appetite regulation in healthy individuals [48]. An increase in basal metabolic rate as a result of exposure to hypoxia is also a factor that might have contributed to the negative energy balance and body mass reductions observed in both groups. While we did not perform the metabolic rate measurements, previous reports suggest that energy intake requirements at high altitudes may be higher as a consequence of increased basal metabolic rate [49].

While the results of this study extend our understanding of hormonal appetite regulation during combined exercise and hypoxia a few limitations of this study need to be addressed. First, the present study investigated the effects of normobaric rather than hypobaric hypoxia. Even though there are no known effects of pressure *per se* on hormonal appetite regulation, recent reports suggest that, for certain physiological functions, normobaric and hypobaric hypoxia do in fact induce somewhat different responses [50]. Second, due to the relatively small sample size the results should be interpreted with caution and it is warranted that the hypothesis of a synergistic effect of hypoxia and exercise is tested using higher number of participants. Third, to determine the potential orexigenic effects of combined hypoxia and exercise we measured resting and postprandial responses of total ghrelin rather than its acylated (active) form, suggested to mainly underlie the appetite inducing effects of ghrelin [13]. Finally, the dose of both, hypoxia and exercise should be taken into account when interpreting the findings of the present study. Based on previous reports indicating profound reductions in appetite as a result of exposures to extreme altitudes (>5000 m) [3], more severe hypoxia would probably exert larger effects on the measured appetite markers than those observed in the present study.

## Conclusion

While some minor metabolic benefits of exercise training during normobaric hypoxic confinement were noted (i.e. decreased postprandial glucose response and reduced cholesterol levels), the findings of this study do not support the hypothesis that adding exercise training to hypoxic exposure significantly alters appetite regulation or improves metabolic outcomes. In addition, our findings indirectly indicate that glucose uptake and gut permeability are unaltered following prolonged hypoxia with or without exercise. Based on the current inconsistencies in the literature and given the escalating prevalence of obesity and metabolic dysfunctions, future well-controlled and powered dose-response trials are warranted to ultimately determine the potential of combining hypoxia and exercise for reducing appetite and improving cardio-metabolic risk factors in both clinical populations and healthy humans.
